# Serological diagnosis of toxoplasmosis: evaluation of the commercial test *recom*Line *Toxoplasma* IgG immunoblot (Mikrogen) based on recombinant antigens[Fn FN1]

**DOI:** 10.1051/parasite/2022050

**Published:** 2022-11-09

**Authors:** Vincent Jean-Pierre, Julien Miozzo, Hélène Fricker-Hidalgo, Cécile Garnaud, Marie Gladys Robert, Hervé Pelloux, Marie-Pierre Brenier-Pinchart

**Affiliations:** 1 Parasitology-Mycology, Grenoble Alpes University Hospital 38000 Grenoble France; 2 TIMC, CNRS, Université Grenoble Alpes 38000 Grenoble France

**Keywords:** Toxoplasmosis, Serology, Immunoblot, Western blot, Seroconversion

## Abstract

*Background*: IgG detection to determine immune status to *Toxoplasma gondii* infection and seroconversion mainly relies on ELISA techniques and, if necessary, on a confirmatory test, Western blot. This study evaluated the performance of the *recom*Line *Toxoplasma* IgG immunoblot (IB-*recom*Line) (Mikrogen) as a confirmatory test on a large number of sera. A total of 171 sera were selected (113 patients) and had previously been analyzed by two ELISA tests, ARCHITECT (Abbott) and VIDAS (bioMérieux) ± LDBIO-Toxo II IgG Western blot (WB-LDBIO) (LDBio). The sera were classified into three groups: group 1 included 50 sera without difficulty in interpreting the IgG results (patients with documented past infection or uninfected); group 2 included 47 sera with difficulty in interpreting the ELISA results; and group 3 included 74 sequential sera from 25 pregnant women with seroconversion. *Results*: In group 1, overall IgG agreements were 94% and 90% with ARCHITECT and VIDAS, respectively. In group 2, low agreement was observed between IB-*recom*Line and WB-LDBIO, with eight false-positive and 13 false-negative results. In group 3, 4/13 seroconversions were detected earlier with IB-*recom*Line compared to other tests. *Conclusions*: IB-*recom*Line allowed for earlier diagnosis of toxoplasmic seroconversion compared to both ELISA tests and WB-LDBIO but led to insufficient performance to confirm the immune status when ELISA results were discordant or equivocal.

## Abstract (fr)

*Contexte* : La détection des IgG pour déterminer le statut immunitaire vis-à-vis de l’infection à *Toxoplasma gondii* et de la séroconversion repose principalement sur les techniques ELISA et, si nécessaire, sur un test de confirmation, le Western blot. Cette étude a évalué les performances de l’immunoblot *recom*Line *Toxoplasma* IgG (IB-*recom*Line) (Mikrogen) en tant que test de confirmation sur un grand nombre de sérums. Un total de 171 sérums ont été sélectionnés (113 patients) et ont été préalablement analysés par deux tests ELISA, ARCHITECT (Abbott) et VIDAS (bioMérieux) ± LDBIO-Toxo II IgG Western blot (WB-LDBIO) (LDBio). Les sérums ont été classés en trois groupes : le groupe 1 comprenait 50 sérums sans difficulté d’interprétation des résultats IgG (patients avec antécédents d’infection documentée ou non infectés); le groupe 2 comprenait 47 sérums avec des difficultés d’interprétation des résultats ELISA; le groupe 3 comprenait 74 sérums séquentiels de 25 femmes enceintes séroconverties. *Résultats* : Dans le groupe 1, les concordances globales des IgG étaient respectivement de 94 % et 90 % avec ARCHITECT et VIDAS. Dans le groupe 2, une faible concordance a été observée entre IB-*recom*Line et WB-LDBIO, avec huit faux positifs et 13 faux négatifs. Dans le groupe 3, 4/13 séroconversions ont été détectées plus tôt avec IB-*recom*Line par rapport aux autres tests. *Conclusions* : IB-*recom*Line a permis un diagnostic plus précoce de la séroconversion toxoplasmique par rapport aux tests ELISA et WB-LDBIO, mais a conduit à des performances insuffisantes pour confirmer le statut immunitaire lorsque les résultats ELISA étaient discordants ou équivoques.

## Introduction

Toxoplasmosis is a widespread parasitic zoonosis caused by *Toxoplasma gondii*, an obligate intracellular protozoan parasite that infects approximately 25%–30% of the world’s population [25]. This infection is usually asymptomatic or accompanied by self-limited signs in immunocompetent patients, but can result in severe forms in immunocompromized patients and complications in newborns when a mother acquires her first *T. gondii* infection during pregnancy (congenital toxoplasmosis) [4, 34]. Since the clinical signs of toxoplasmosis are non-specific, diagnosis of this infection is primarily based on serology which relies on the detection of both specific anti-*T. gondii* IgM and IgG antibodies [8, 31].

Determining the immune status is essential in three main situations: pregnant women, immunocompromized patients and transplant recipients or donors [26]. For pregnant women, detection of specific IgG antibodies without IgM detection at the beginning of pregnancy indicates a past infection and rules out the risk of a congenital primary infection [21]. Conversely, negative IgG serology implies compliance with hygiene recommendations ± follow-up of pregnant women according to national guidelines [12]. For immunocompromized patients (HIV infection, immunosuppressive treatments, organ and hematopoietic stem cell transplantations, etc.), it is essential to diagnose and treat primary infection or toxoplasmic reactivation to avoid life-threatening encephalitis, myocarditis, pneumonitis or disseminated infection [5]. Finally, it is essential to identify the risk of mismatch of a positive organ donor (D +) to a negative organ recipient (R −) and prevent disseminated toxoplasmosis in the recipient.

Although IgM antibodies are the first serological markers to become positive following *T. gondii* infection, the detection of IgM alone is not conclusive [32]. Confirmation of primary infection is indicated by the appearance of anti-*T. gondii*-specific IgG in a previously non-immune patient (seroconversion). Usually, specific IgGs appear one to three weeks after IgM and are mostly detected with enzyme-linked immunosorbent assays (ELISA). These indirect immunoenzymatic tests use a mixture of antigens and are performed on automated analyzers [7]. However, interpreting low or equivocal ELISA IgG titers or discordance between ELISA tests can be difficult and requires further testing [32]. The Sabin–Feldman dye test is the historical gold standard method using live *T. gondii* [24]. However, due to its high price and its fastidious use, the dye test is not commercially available and only used today in a few specialized laboratories [14]. The LDBIO-Toxo II IgG Western blot (WB-LDBIO) (LDBio Diagnostics, Lyon, France), based on natural *T. gondii* antigens, is currently recommended as a confirmatory test [32]. The *recom*Line *Toxoplasma* IgG immunoblot (IB-*recom*Line) (Mikrogen Diagnostik, Neuried, Germany), based on recombinant *T. gondii* antigens, is another qualitative assay that is still little used [22, 30].

The objective of this work was to assess whether IB-*recom*Line could be used as a complementary test to confirm the absence or presence of anti-*T. gondii*-specific IgG when the results of the ELISA tests do not allow for clear biological interpretation, like WB-LDBIO. For this purpose, we evaluated the diagnostic performance of IB-*recom*Line for the detection of IgG in 171 selected and characterized human sera from three different clinical and biological settings: sera without difficulty in biological interpretation, sera that lead to difficulties in biological interpretation, and sequential sera from infected mothers with proven *T. gondii* seroconversion.

## Material and methods

### Ethics

This non-interventional, monocentric, retrospective study involving data and samples from human participants was carried out in the Grenoble Alpes University Hospital according to current French regulations. The principal investigator (Dr. Marie-Pierre Brenier-Pinchart, MD, PhD) has signed a commitment to comply with Reference Methodology No. MR004 issued by the French Authorities (CNIL). All subjects were informed and were not opposed to the research; written consent for participation was not required for this study in accordance with national legislation and institutional requirements. The raw data supporting the conclusions of this article will be made available by the authors in respect of the General Data Protection Regulation, without undue reservation.

### Patients and sera

A total of 171 sera collected from 113 patients were included and stored at −20 °C for a few months up to five years (between May 2016 to April 2021) [3]. The study population included 92 women (81%) and 21 men (19%). The mean age was 42 years (range: 21–92 years).

These sera were classified into three groups based on previous results obtained in routine diagnosis and were retrospectively analyzed using IB-*recom*Line to examine the performance of the test in various diagnostic conditions. Group 1 included 50 sera without difficulty in biological interpretation with ELISA tests in routine diagnosis. Among these sera: 16 had a serological profile indicating a non-immune status to *T. gondii* (negative results with ELISA techniques) and 34 corresponded to a past infection where the individuals were immunized against *T. gondii* (positive or equivocal results with ELISA techniques). Among these 34 sera, three were equivocal in ELISA but were considered well-defined sera based on previous positive sera for each of these three patients.

**Figure 1:**
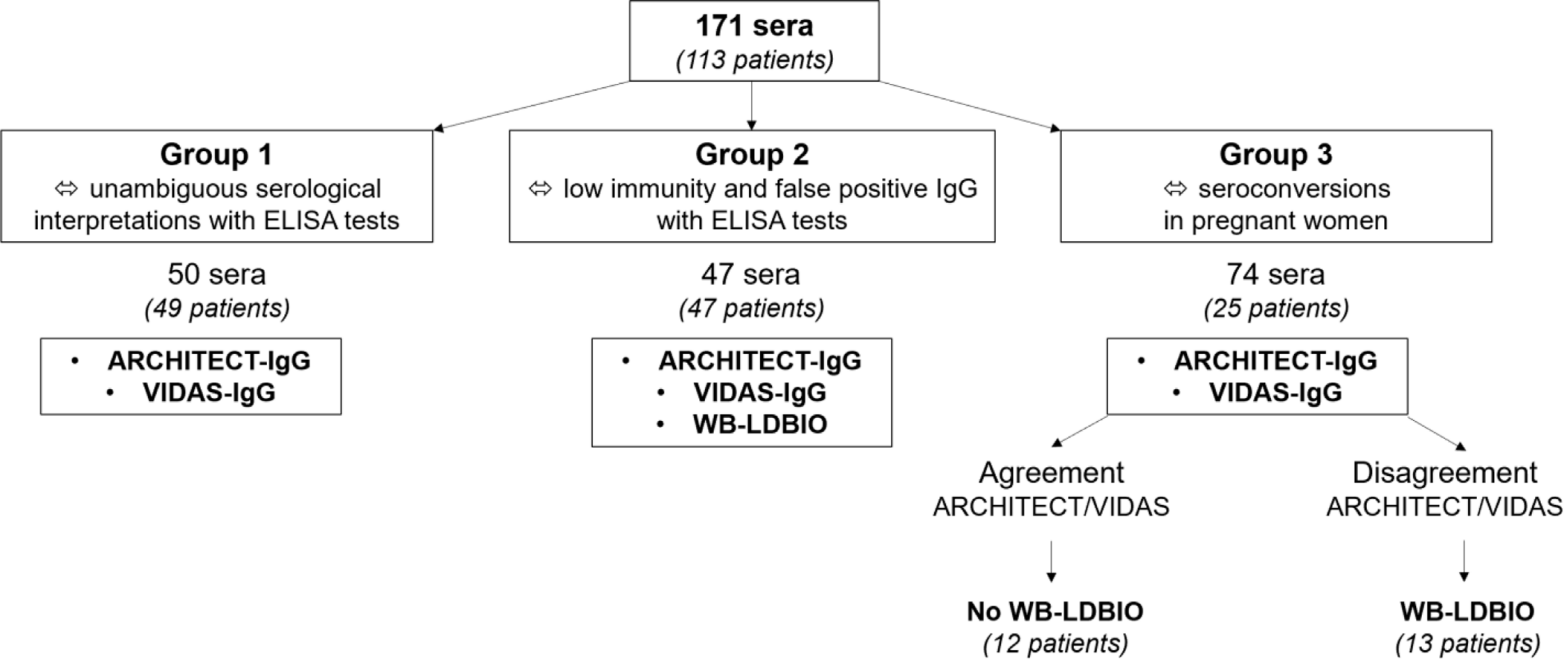
Classification of sera into three groups and IgG detection techniques performed on each category of sera.

Group 1 was designed to assess the IgG agreement between IB-*recom*Line and ELISA assays in unambiguous cases. In contrast, group 2 included 47 sera with equivocal or discordant IgG results with ELISA techniques. Sera were considered discordant in the following situations: serum negative in one ELISA technique and positive in the second one; serum equivocal in one ELISA technique and positive (or negative) in the second one. Group 2 was designed to assess the IgG agreement between IB-*recom*Line and WB-LDBIO. The objective was to evaluate the IB-*recom*Line capacity to detect low IgG titers in past infection and to resolve discrepant results between ELISA assays to unambiguously determine the immune status of the selected patients. Finally, group 3 included 74 sequential sera from 25 pregnant women with proven *T. gondii* seroconversion (one to five consecutive sera for each patient). Group 3 was designed to compare capabilities for early detection of toxoplasmic seroconversion between IB-*recom*Line and WB-LDBIO in pregnant women (Fig. 1).

### IgG serological tests

All commercial tests were performed in the Grenoble Alpes University Hospital (France) Parasitology Mycology unit and interpreted according to the manufacturer’s instructions.

#### Automated immunoassays

The ARCHITECT^®^ Toxo IgG test (ARCHITECT-IgG) (Abbott Laboratories, Wiesbaden, Germany) is a fully automated chemiluminescent microparticle immunoassay for the quantitative determination of IgG antibodies against *T. gondii* in human serum using recombinant antigens [SAG1 (= p30), GRA8 (= p35)]-coated microparticles [29]. The VIDAS^®^ Toxo IgG II test (VIDAS-IgG) (bioMérieux, Marcy l’Étoile, France) is a fully automated ELISA for the quantitative determination of IgG antibodies against *T. gondii* in human serum using native antigen SAG1 (= p30)-coated microparticles [27].

Both automated ELISA tests were previously performed on the 171 selected sera (Fig. 1). According to the manufacturer, ARCHITECT-IgG results are positive if ≥3 IU/mL, negative if <1.6 IU/mL, and equivocal if ≥1.6 and <3 IU/mL. VIDAS-IgG results are positive if ≥8 IU/mL, negative if <4 IU/mL, and equivocal if ≥4 and <8 IU/mL; ARCHITECT-IgM results are positive if ≥0.6, negative if <0.5, and equivocal if ≥0.5 and <0.6. VIDAS-IgM results are positive if ≥0.65, negative if <0.55, and equivocal if ≥0.55 and <0.65.

#### LDBIO-Toxo II IgG Western blot (WB-LDBIO)

WB-LDBIO (LDBio Diagnostics, Lyon, France) is a qualitative *in vitro* test for the detection of IgG antibodies against *T. gondii* in human serum. Once separated by electrophoresis, natural *T. gondii* antigens of different molecular weights 30 kDa (= p30), 31 kDa, 33 kDa, 40 kDa and 45 kDa, are bound by electroblotting to the surface of a nitrocellulose membrane [17]. According to the manufacturer, WB-LDBIO is considered positive when at least three specific bands including p30 are present on the strip [6, 18].

Since WB-LDBIO showed perfect agreement with the dye test gold standard technique, it was used as the confirmatory test in this study [15]. For group 1, when sera gave equivocal results, WB-LDBIO tests were not performed to confirm immune status because the IgG titers were positive in a previous serum sample for these patients. For group 2, all serologies were prospectively confirmed with WB-LDBIO to interpret serological profiles. For group 3, WB-LDBIO was retrospectively performed in each serum from seroconversion in the case of qualitative discordance between ELISA techniques (ARCHITECT-IgG and VIDAS-IgG) (Fig. 1).

#### *Recom*Line *Toxoplasma* IgG immunoblot (IB-*recom*Line)

The IB-r*ecom*Line test (Mikrogen Diagnostik, Neuried, Germany) is another qualitative *in vitro* test for detecting IgG antibodies against *T. gondii* in human serum. Highly purified recombinant *T. gondii* antigens [ROP1c (= p66), GRA7 (= p29), GRA8 (= p35), SAG1 (= p30), MAG1 (= p65, p68), GRA1 (= p24), rSAG1 (= p30, low concentration)] are fixed on nitro-cellulose membrane test strips. The intensity of the cut-off band allowed the reactivity of each antigen band to be assessed. Only the bands for which the intensity was higher than or equal to the cut-off band were considered positive. ROP1c counted for 1 point, MAG1 counted for 2 points, GRA1, GRA7, GRA8 and rSAG1 counted for 4 points each, p30 counted for 6 points. According to the manufacturer, IB-*recom*Line is considered positive when the total number of points reported by the positive bands (total) is ≥ 6, negative when the total is ≤ 3, and equivocal when the total is 4 or 5 [22]. Analysis of the test strips was computer-assisted using the test strip analysis software *recom*Scan.

### Data analysis

IB-*recom*Line sensitivity (Se) and specificity (Sp) were estimated by comparing IB-*recom*Line qualitative results (negative, equivocal or positive) with the overall biological interpretation given by ELISA assays (ARCHITECT and VIDAS) ± WB-LDBIO results ± patient serological anteriority.

The value of each parameter was calculated twice: with IB-*recom*Line equivocal results considered negative or positive (Table 2).

A minor discrepancy was defined as an equivocal result in IB-*recom*Line while the biological interpretation was either negative or positive. A major discrepancy was defined as a negative result in IB-*recom*Line while the biological interpretation was positive and *vice versa*.

Overall IgG agreements (and Cohen’s kappa values) between IB-*recom*Line and ELISA assays in group 1, and between IB-*recom*Line and WB-LDBIO in group 2, were calculated as follows: agreement = (number of concordant samples/number of tested samples on both assays) × 100%. Agreement with kappa values of 0.21 to 0.40 was considered low, 0.41 to 0.60 was considered moderate, 0.61 to 0.80 was considered substantial, and 0.81 to 1.00 was considered almost perfect [16].

In group 3, the positivity rate for each technique (ARCHITECT, VIDAS, WB-LDBIO, IB-*recom*Line) was the percentage of equivocal and positive IgG results among the first sera in which IgG was detected by at least one test.

## Results

### High agreement between IB-*recom*Line and ELISA assays in group 1

Results of IgG assays for group 1 are provided in Table 1. Group 1 included unambiguous cases without difficulty in biological interpretation: 16 sera without IgG (no immunization) and 34 sera with low or high titers of IgG (past infection) in ELISA assays. Among the 16 sera IgG negative with both ARCHITECT and VIDAS: 15 were also negative with IB-*recom*Line and one sample was equivocal and represented a minor discrepancy. Among the 34 sera with low or high titers of IgG: a) three were equivocal in VIDAS ± ARCHITECT (with previous positive sera) and were positive with IB-*recom*Line, and b) 31 were IgG positive with both ARCHITECT + VIDAS; 30 were also positive with IB-*recom*Line while one was equivocal and represented a minor discrepancy.

**Table 1:** IB-recomLine IgG results of the 97 characterized sera of groups 1 and 2.

	Number of sera	Biological interpretation	IB-*recom*Line result (number of samples)			WB-LDBIO result	ARCHITECT-IgG result	VIDAS-IgG result
			−	eq	+			
Group 1 (50 sera)	16	Negative	15	1*	0	NP	−	−
	34	Positive	0	0	1	NP	**eq**	**eq**
			0	0	2	NP	+	**eq**
			0	1*	30	NP	+	+
Group 2 (47 sera)	16	Negative	4	1*	0	−	−	eq
			2	2*	1**	−	eq	−
			1	2*	1**	−	+	−
			1	1*	0	−	eq	eq
	31	Positive	0	0	1	+	−	eq
			2**	1*	0	+	eq	−
			1**	0	0	+	−	+
			1**	0	1	+	+	−
			1**	3*	12	+	eq	eq
			1**	1*	3	+	eq	+
			1**	1*	1	+	+	eq

+: positive result; −: negative result; eq: equivocal result; **eq: equivocal result with previous positive sera**; NP: test not performed; *: minor discrepancy with biological interpretation; **: major discrepancy with biological interpretation.

In total, among the 33 IB-*recom*Line-positive cases, the p30 band was consistently present. The GRA7 and GRA8 bands were frequently present (64% and 70% of cases, respectively), followed by the MAG1 band (45%), the GRA1 and rSAG1 bands (36% each) and the ROP1c that appeared less frequently (24%).

Overall IgG agreement was good with almost perfect agreement between IB-*recom*Line and ARCHITECT: 94% (kappa = 0.87), and substantial agreement between IB-*recom*Line and VIDAS: 90% (kappa = 0.8). Depending on whether equivocal results of IB-*recom*Line were considered negative or positive, Se values were 97.1% [84.7–99.9] and 100% [89.7–100] and Sp values were 100% [79.4–100] and 93.8% [69.8–99.8], respectively (Table 2).

**Table 2:** IgG titers, IB-*recom*Line sensitivity, specificity and overall agreement between IB-*recom*Line and other assays.

Serum group (number of sera)		ELISA mean IgG levels ± SD (IU/mL)		IB-*recom*Line sensitivity (%) [95% CI]		IB-*recom*Line specificity (%) [95% CI]		IgG overall agreement (%) (kappa value) between IB-*recom*Line and other assays		
		ARCHITECT	VIDAS	Equivocal IB results = negative	Equivocal IB results = positive	Equivocal IB results = negative	Equivocal IB results = positive	ARCHITECT	VIDAS	WB-LDBIO
	*Negative*	<1.6	<4							
	*Equivocal*	[1.6–2.9]	[4–7]							
	*Positive*	≥3	≥8							
1 (*n =* 50)		51.4 ± 112.1	95.1 ± 102.9	97.1 [84.7–99.9]	100 [89.7–100]	100 [79.4–100]	93.8 [69.8–99.8]	94 (0.87)	90 (0.8)	NP
2 (*n* = 47)		2.9 ± 3.5	6.7 ± 2.8	58.1 [39.1–75.5]	77.4 [58.9–90.4]	87.5 [61.7–98.5]	50 [24.7–75.4]	NP	NP	55.3 (0.27)

SD: standard deviation; CI: confidence interval; NP: not performed

### Low agreement between IB-*recom*Line and WB-LDBIO in group 2

Results of IgG assays for group 2 are provided in Table 1. Group 2 included 47 sera with equivocal results and/or qualitative discordances between ELISA assays; therefore, a confirmatory technique was needed to conclude and to propose a biological interpretation. IB-*recom*Line results were compared to those obtained with the WB-LDBIO currently used as a reference confirmatory test in our laboratory and both overall IgG and kappa agreements were low: 55.3% (kappa = 0.27). Depending on whether equivocal results of IB-*recom*Line were considered negative or positive, Se values were 58.1% [39.1–75.5] and 77.4% [58.9–90.4], and Sp values were 87.5% [61.7–98.5] and 50% [24.7–75.4], respectively (Table 2).

In total, there were 12 minor discrepancies and nine major discrepancies. Among the eight IB-*recom*Line false-positive cases, the GRA8 band was present in six cases (75%), the GRA7 band was present twice, and the ROP1c, p30 and GRA1 bands were present only once. Furthermore, among these eight sera, six were also false-positive in ARCHITECT and negative in VIDAS; one was equivocal in both ARCHITECT and VIDAS and one other was equivocal in VIDAS and negative in ARCHITECT.

**Figure 2:**
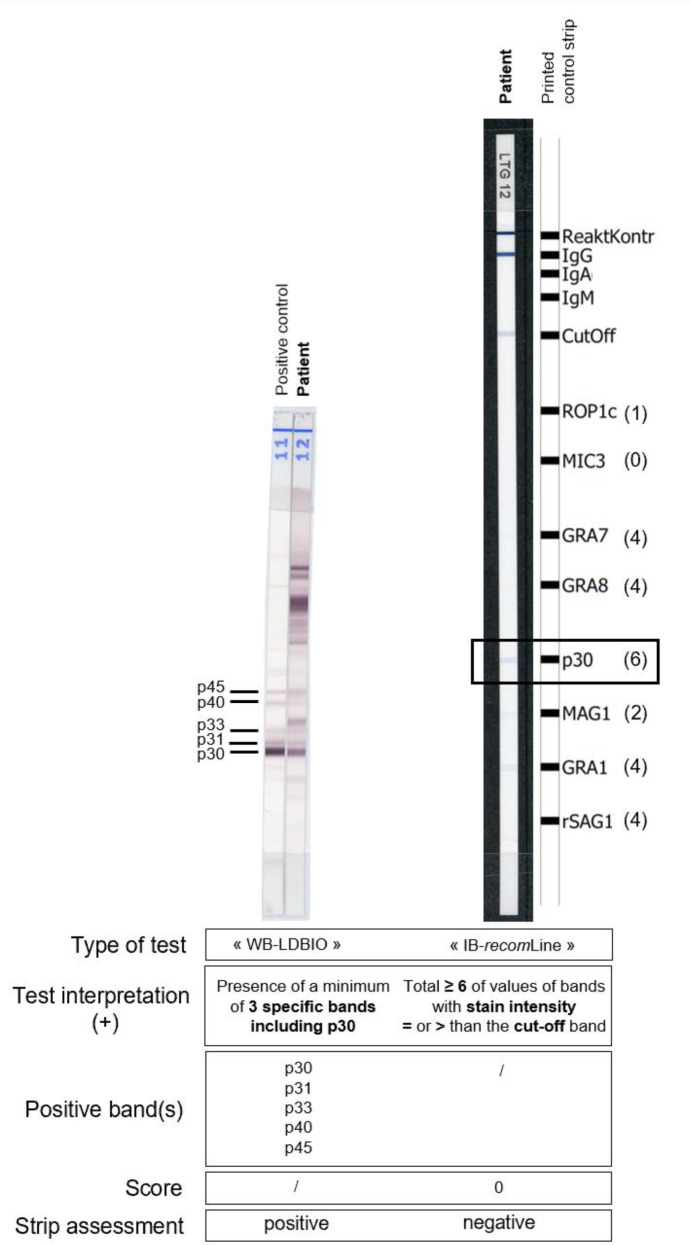
Example of a false-negative result with IB-*recom*Line compared to WB-LDBIO. The number of points reported by positive bands with IB-*recom*Line is specified in parentheses behind each antigen*.*

There were also 13 IB-*recom*Line false-negative results that corresponded to sera from patients with chronic *T. gondii* infection with IgG that persisted at residual titers not detected with IB-*recom*Line. Among them, we visually identified the bands whose intensity appeared slightly lower than the cut-off band and were considered negative by the *recom*Scan software. The p30 and GRA1 bands were detectable with visual reading, but not quantifiable with *recom*Scan software in nine (69%) and seven cases (54%), respectively followed by the GRA8 (38%), GRA7 (31%) and MAG1 (23%) bands. An example of a false-negative result with the IB-*recom*Line is described in Figure 2. In this figure, WB-LDBIO was positive with all specific IgG bands. However, IB-*recom*Line was negative because the intensity of the p30 band was not sufficient to be considered positive by the *recom*Scan software and the total score of the bands was 0.

### Precocity of seroconversion detection with IB-*recom*Line compared to other techniques (group 3)

WB-LDBIO was retrospectively performed when at least one serum for each seroconversion showed discordance between ARCHITECT-IgG and VIDAS-IgG results (13/25 seroconversion sequences). Antibody kinetics (IgM and IgG) in these 13 seroconversions are provided in Table 3. We examined the positivity rate (equivocal or positive results) of serological techniques for the first sera in which IgG was detected by at least one assay. We showed that IB-*recom*Line was positive in 92.3% of the cases (12/13), followed by ARCHITECT-IgG (61.5%, 8/13), WB-LDBIO (46.2%, 6/13) and VIDAS-IgG (15.4%, 2/13). The positivity of IB-*recom*Line preceded all other tests in 4/13 cases while ARCHITECT-IgG, WB-LDBIO and VIDAS-IgG were still negative. In contrast, VIDAS-IgG was the last assay to become positive in 7/13 cases, while all other tests were either equivocal or positive. A total of 7/13 seroconversions were detected earlier with IB-*recom*Line compared to WB-LDBIO (four positive and three equivocal with IB-*recom*Line). A total of 2/13 seroconversions were detected later with IB-*recom*Line than with WB-LDBIO and 4/13 seroconversions were detected simultaneously by both techniques.

**Table 3:** Antibody kinetics (IgM and IgG) in 13 cases of women with proven seroconversion.

		Toxo IgM assay results		Toxo IgG assay results (IU/mL)			
Case no.	Available sera (days)	ARCHITECT (index)	VIDAS (index)	ARCHITECT	VIDAS	WB-LDBIO	IB-*recom*Line
1	D0	0.05	0.04	0.1	0	Negative	Negative
	D32	**3.95**	**1.66**	**4.1**	1	**Positive**	**Positive**
	D40	**3.28**	**1.75**	**13.1**	**8**	**Positive**	**Positive**
2	D0	0.04	0.07	0.1	0	Negative	Negative
	D102	0.39	0.47	**5.8**	2	**Positive**	**Positive**
	D109	0.42	0.5	**9.2**	7	**Positive**	**Positive**
3	D0	**1.22**	**0.82**	2.4	0	Negative	**Positive**
	D9	**1.21**	**0.95**	**9.5**	4	**Positive**	**Positive**
	D11	**1.11**	**1**	**12.4**	7	**Positive**	**Positive**
4	D0	**5.31**	**3.36**	2	2	Negative	**Positive**
	D7	**4.02**	**3.06**	**5.2**	**8**	Negative	**Positive**
	D54	**1.99**	**1.66**	**75.6**	**123**	**Positive**	**Positive**
5	D0	**1.44**	**0.84**	0.4	0	Negative	Negative
	D7	**1.63**	**1.02**	1.1	2	Negative	**Positive**
	D18	**1.61**	**1.11**	2.9	**8**	**Positive**	**Positive**
6	D0	**3.92**	**2.15**	1.7	0	**Positive**	Negative
	D7	**3.51**	**2.16**	2.4	0	**Positive**	Equivocal
	D18	**3.5**	**2.05**	**5.8**	6	**Positive**	**Positive**
	D183	**0.71**	**11**	**6.2**	7	**Positive**	Equivocal
7	D0	0.07	0.14	0	0	Negative	Negative
	D41	**3.37**	**2.46**	**5**	3	Negative	Equivocal
	D44	**3.2**	**2.62**	**8**	6	**Positive**	**Positive**
	D87	**3.7**	**2.51**	**141.8**	**106**	**Positive**	**Positive**
8	D0	**7.18**	**4.5**	**6.9**	2	**Positive**	**Positive**
	D8	**8.35**	**4.83**	**23.9**	**14**	**Positive**	**Positive**
9	D0	**11.24**	**5.4**	**5**	6	**Positive**	Equivocal
	D144	**3.55**	**3.07**	1.9	2	**Positive**	Negative
10	D0	**1.38**	**0.92**	0.1	0	Negative	**Positive**
	D9	**2.55**	**1.92**	0.7	0	**Positive**	**Positive**
	D22	**2.22**	**1.52**	**3.5**	1	**Positive**	**Positive**
	D29	**1.83**	**1.36**	**6**	4	**Positive**	**Positive**
11	D0	0.59	0.48	1.4	0	Negative	Equivocal
	D35	**4.26**	**3.64**	**20.7**	0	**Positive**	**Positive**
	D43	**6.46**	**4.58**	**47.7**	2	**Positive**	**Positive**
	D85	**2.63**	**2.62**	**259.2**	**119**	**Positive**	**Positive**
12	D0	**6.62**	**4.79**	1.1	0	Negative	Equivocal
	D28	**4.75**	**3.9**	1.7	1	**Positive**	**Positive**
	D35	**4.62**	**3.98**	1.6	1	**Positive**	**Positive**
	D63	**3.65**	**3.05**	2.4	1	**Positive**	Equivocal
	D122	**2.53**	**2.17**	2.7	1	**Positive**	Equivocal
13	D0	**5.81**	**4.17**	**5.8**	4	**Positive**	**Positive**

D: day; shaded boxes indicate equivocal results; values in boldface represent positive results.

**Figure 3:**
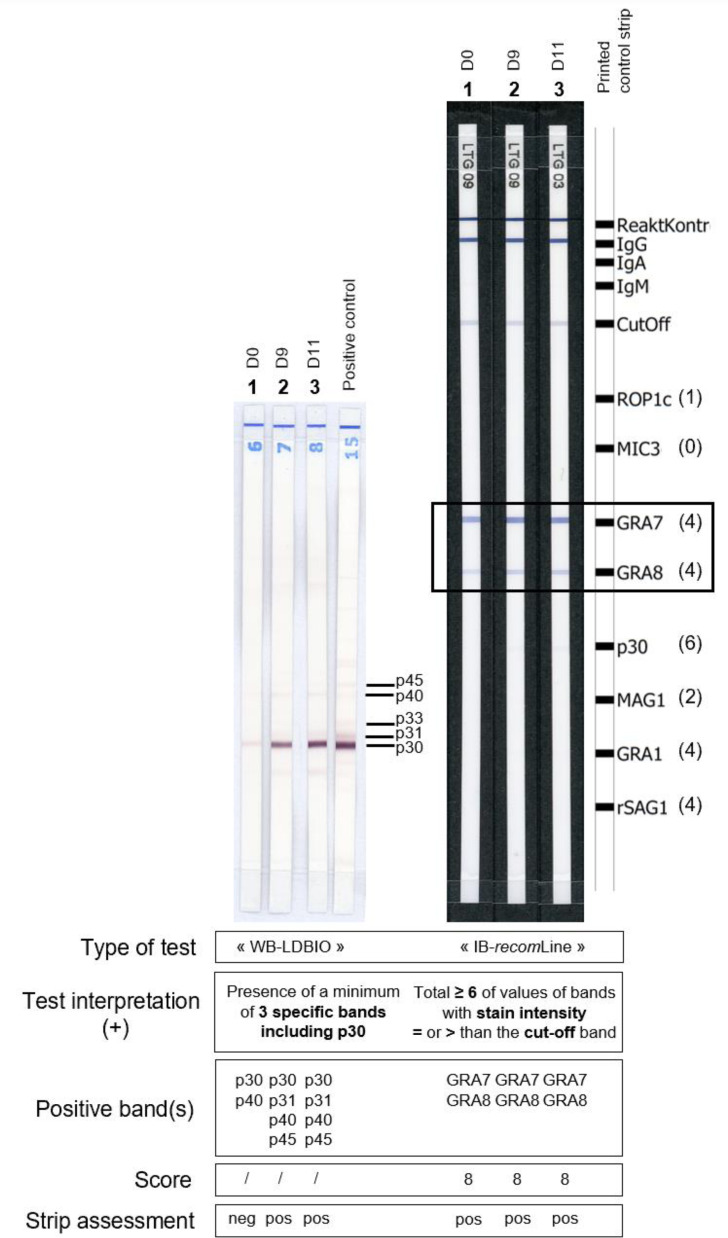
Example of toxoplasmic seroconversion with IgG detected earlier with IB-*recom*Line compared to WB-LDBIO. In this sequence of three sera belonging to the same patient: serum 1 corresponded to “day 0”, serum 2 to “day 9”, and serum 3 to “day 11”. The number of points reported by positive bands with IB-*recom*Line is specified in parentheses. Neg = negative result; pos = positive result.

Among the seven IB-*recom*Line results allowing earlier seroconversion diagnosis compared to WB-LDBIO, the GRA8 and GRA7 bands were present in four cases each and the p30 and MAG1 bands were present once each. Figure 3 provides an example of toxoplasmic seroconversion with IgG detected earlier with the IB-*recom*Line compared to the WB-LDBIO. Serum 1 was considered IgG negative with the WB-LDBIO technique because only the p30 and p40 bands were slightly positive. However, the same serum was considered IgG positive with the IB-*recom*Line technique because both GRA7 and GRA8 bands were positive and reported a total of 8 points to the strip.

In addition, when ARCHITECT-IgG and VIDAS-IgG were concordant, WB-LDBIO was not performed and IB-*recom*Line was consistently concordant with ELISA tests (12/25 seroconversion sequences) (data not shown).

## Discussion

The diagnosis of toxoplasmosis in immunocompetent patients relies mainly on the detection of anti-*T. gondii* antibodies. While in most cases, the use of ELISA tests is sufficient to determine the immune status of patients, the clinical interpretation of the results of first-line techniques may require the use of a confirmatory technique [32]. WB-LDBIO, based on natural *T. gondii* antigens, is currently recommended as a reference confirmatory test thanks to its high sensitivity (99.2%) and its perfect specificity (100%) [6]. Unlike WB-LDBIO, IB-*recom*Line, based on recombinant antigens, remains seldom described in the literature. To our knowledge, this study is the first that has evaluated the diagnostic performance of IB-*recom*Line as a confirmatory serological test for the detection of IgG. We challenged IB-*recom*Line with several groups of characterized sera to determine its ability to respond to different clinical and biological settings in which it could be used.

IB-*recom*Line was evaluated for its ability to detect low levels of formerly synthesized IgG. In our study, IB-*recom*Line exhibited unsatisfactory analytical performance with too many false-negative results (13/47, including seven major discrepancies). In a large majority of these misdiagnosed sera, the intensity of the p30 (SAG1) band was close to being interpreted as positive. As this band alone rates for 6 points, it would have made IB-*recom*Line positive. This apparent lack of sensitivity in detecting residual IgG titers compared to WB-LDBIO could be explained by the nature of p30 antigen that differs between these two assays. Natural p30 antigen of WB-LDBIO might enable more sensitive diagnosis of chronic *T. gondii* infection than recombinant p30 antigen of IB-*recom*Line. Therefore, the use of IB-*recom*Line as a confirmatory test could result in unnecessary and expensive serological follow-up in pregnant woman, for instance in the French context [28]. It could also misdiagnose seropositive organ donors, which could lead to life-threatening consequences with transmission of toxoplasmosis to seronegative transplant recipients (D+/R−) [7].

The lack of sensitivity of IB-*recom*Line to detect low IgG titers in patients with past infection contrasted sharply with its ability to detect low IgG titers early after primary infection. In our study, IB-*recom*Line was the most sensitive technique for detecting the appearance of newly synthesized IgG in pregnant women followed by ARCHITECT-IgG, WB-LDBIO, and VIDAS-IgG. In fact, among the 13 sera where seroconversion was detected, IB-*recom*Line was positive (or equivocal) 12 times. Until now, WB-LDBIO was considered to be the most sensitive commercial technique [19]. Armengol et al. showed that the median durations before positive IgG detection were shortened by 13 days and more than 20 days with the use of WB-LDBIO compared to ARCHITECT-IgG and VIDAS-IgG, respectively [1]. As shown by Armengol et al. and many previous reports, we found that IgG detection with VIDAS-IgG was delayed in 7/13 seroconversions, being the last technique allowing detection of IgG in pregnant women in our study [1, 9, 20]. To date, there is only one study in the literature that has focused on IB-*recom*Line and more particularly on the value of the different recombinant antigens it uses to improve the diagnosis of acute toxoplasmosis during pregnancy [22]. Pfrepper et al. showed that IgG antibodies against GRA7 and GRA8 were exclusively present at the beginning of the IgG response, and those against GRA7 were the most present in patients with recent seroconversion [22]. Our study strengthened these findings showing that most of the time, GRA7 and GRA8 were the bands that appeared first following an early seroconversion, contrary to p30 band that appeared only once. Therefore, GRA8 and GRA7 bands would allow earlier detection of newly synthetized IgG antibodies. These observations could explain the precocity of IB-*recom*Line compared to other techniques that use neither GRA7 nor GRA8, such as VIDAS-IgG. Providing a *T. gondii* seroconversion diagnosis before other tests, IB-*recom*Line would enable earlier therapeutic intervention that may reduce the risk of brain lesions in infected newborns even though it would have little or no impact on the fetomaternal transmission rate [10, 11, 13, 33].

Interestingly, in most studies, p30 is known to be one of the most immunogenic antigens following acute seroconversion, possibly due to its position on the surface membrane of the tachyzoite stage of *T. gondii* [2, 23]. However, our study showed a lack of reactivity of IgG antibodies against p30 in the early stages of infection, as already described by Pfrepper et al. [22]. This last result reinforces the hypothesis that the formulation of recombinant p30 antigen of IB-*recom*Line is probably not appropriate.

Equivocal IgG results and/or discordances between ELISA tests may correspond either to low titers of IgG in case of past infection or to the beginning of seroconversion, but may also correspond to false-positive IgG results. We showed that 7/8 false-positive results obtained with the ARCHITECT-IgG are also false-positive with IB-*recom*Line. Similarly to IB-*recom*Line and unlike VIDAS-IgG and WB-LDBIO, ARCHITECT-IgG uses the GRA8 recombinant antigen to detect anti-*T. gondii*-specific IgG. Thus, as already shown by Simon et al., we observed that reactivity against the GRA8 antigen led to false-positive results [30]. Simon et al. hypothesize that past contact with *Neospora caninum* and *Hammondia hammondi*, two parasites close to *T. gondii,* could explain false-positive results with ARCHITECT-IgG and IB-*recom*Line, mostly due to cross-reaction with GRA8 (used by both ARCHITECT and IB-*recom*Line) and also GRA7 to a lesser extent (only used by IB-*recom*Line) [26]. Therefore, IB-*recom*Line could result in false-positive IgG detections and bias the determination of immune status. In total, GRA7 and GRA8 bands were the most frequently detected at the onset of seroconversions, making IB-*recom*Line highly sensitive (group 3), but detection of GRA7 and especially GRA8 bands was also responsible for false-positive results (group 2). Thus, the presumed high sensitivity or earliness of IB-*recom*Line should be interpreted with caution since detection of GRA7 or GRA8 bands may also correspond to false-positive results.

IB-*recom*Line is a manual technique not designed for large-scale screening of toxoplasmosis. Therefore, our study explored its performance as a confirmatory test that has never been done before. The groups selected in this study were composed of few sera, but all these sera were fully characterized by our reference laboratory in toxoplasmosis in Grenoble (France). Digital reading of the bands appeared to be an advantage of IB-*recom*Line over WB-LDBIO, allowing objective evaluation of the bands. However, IB-*recom*Line appeared to have difficulty distinguishing between low positive and negative sera for patients with chronic infection and failed to invalidate false-positive results obtained with ELISA tests, with a risk of serious consequences for patients. Additionally, the presence of a grey zone in the IB-*recom*Line assay, unlike WB-LDBIO, made it impossible to conclude on the immune status of some patients. Its use could be limited to early confirmation of the onset of seroconversion in pregnant women in case of equivocal or discordant results with ELISA tests. In conclusion, although IB-*recom*Line was associated with the quickest detection of seroconversion, it showed insufficient performance to confirm the immune status when ELISA results were discordant or equivocal.

## Abbreviations

ELISA: Enzyme linked immunosorbent assay;
R−: negative recipient;
D+: positive donor;
IB-*recom*Line: *recom*Line *Toxoplasma* IgG immunoblot;
Se: sensitivity;
Sp: specificity;
WB-LDBIO: LDBIO-Toxo II IgG Western blot.
